# Increasing flower species richness in agricultural landscapes alters insect pollinator networks: Implications for bee health and competition

**DOI:** 10.1002/ece3.9442

**Published:** 2022-10-27

**Authors:** Vincent Doublet, Toby Doyle, Isobel Refoy, Sophie Hedges, Claire Carvell, Mark J. F. Brown, Lena Wilfert

**Affiliations:** ^1^ College of Life and Environmental Sciences University of Exeter Penryn UK; ^2^ Institute of Evolutionary Ecology and Conservation Genomics University of Ulm Ulm Germany; ^3^ Department of Comparative Biomedical Sciences The Royal Veterinary College Hatfield UK; ^4^ UK Centre for Ecology & Hydrology Wallingford UK; ^5^ Department of Biological Sciences Royal Holloway University of London Egham UK

**Keywords:** connectance, flower diversity, niche overlap, pollinator networks, restoration, wildflower margins

## Abstract

Ecological restoration programs are established to reverse land degradation, mitigate biodiversity loss, and reinstate ecosystem services. Following recent agricultural intensification that led to a decrease in flower diversity and density in rural areas and subsequently to the decline of many insects, conservation measures targeted at pollinators have been established, including sown wildflower strips (WFS) along field margins. Historically successful in establishing a high density of generalist bees and increasing pollinator diversity, the impact of enhanced flower provision on wider ecological interactions and the structure of pollinator networks has been rarely investigated. Here, we tested the effects of increasing flower species richness and flower density in agricultural landscapes on bee‐plant interaction networks. We measured plant species richness and flower density and surveyed honeybee and bumblebee visits on flowers across a range of field margins on 10 UK farms that applied different pollinator conservation measures. We found that both flower species richness and flower density significantly increased bee abundance, in early and late summer, respectively. At the network level, we found that higher flower species richness did not significantly alter bee species' generality indices, but significantly reduced network connectance and marginally reduced niche overlap across honeybees and bumblebee species, a proxy for insect competition. While higher connectance and niche overlap is believed to strengthen network robustness and often is the aim for the restoration of pollinator networks, we argue that carefully designed WFS may benefit bees by partitioning their foraging niche, limiting competition for resources and the potential for disease transmission via shared floral use. We also discuss the need to extend WFS and their positive effects into spring when wild bee populations are established.

## Graphical abstract

We investigated the potential of wildflower strips to modify plant‐pollinator networks. By comparing networks from wildflower strips and other agricultural field margin habitats, we found that increasing flower species richness significantly reduced network connectance, a measure of network complexity, and marginally reduced bee species' niche overlap, a proxy for insect competition. These findings are of general relevance in ecology and epidemiology, as we provide a new framework for the development of conservation measures where ecological interactions and network structure are taken into consideration beyond the current target of increasing species richness and abundance.

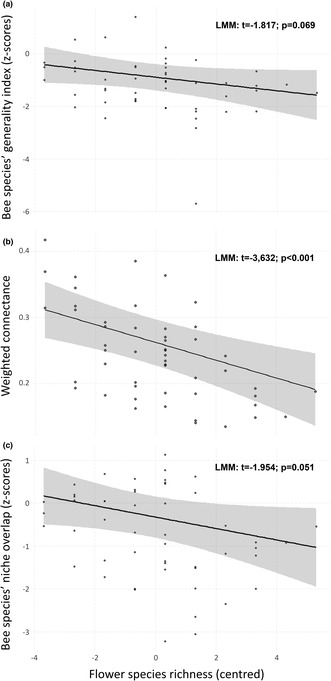

## 1 INTRODUCTION

Anthropogenic disturbance has led to massive biodiversity losses in many ecosystems, and habitat restoration is globally undertaken to re‐establish plant, animal, and bacterial communities (e.g., Barral et al., 2015; Turley et al., 2020). Historically, restoration programs have applied ecological theory to reverse land degradation, mitigate biodiversity loss, and restore valuable ecosystem services (CBD Secretariat, 2010). Designed to protect rare and endangered species, restoration programs have often disregarded the impact on ecological networks and species interactions. However, growing interest in ecological networks within the last decade has prompted the development of a new paradigm of network conservation, that is, restoring the ecological interactions between species (Fraser et al., 2015; Harvey et al., 2017; Menz et al., 2011; Tylianakis et al., 2010; Valiente‐Banuet et al., 2015; Walton et al., 2021).

Agricultural intensification in the second half of the 20th century has dramatically altered rural landscapes in Europe, contributing to the decline of terrestrial insect populations (Hallmann et al., 2017; Seibold et al., 2019; van Klink et al., 2020). To attain higher agricultural productivity, farm sizes increased, crop rotations were simplified, and a significant portion of semi‐natural habitats such as hedgerows and permanent grassland were lost (Baude et al., 2016; Robinson & Sutherland, 2002). The subsequent reduction in flower diversity and abundance and the homogenization of landscapes are considered key threats to insect pollinators such as bees, flies, and butterflies, putting crop pollination and food production at risk (e.g., Hemberger et al., 2021; Powney et al., 2019; Vanbergen & The Insect Pollinators Initiative, 2013). Pollinator‐friendly practices have been implemented to improve habitat quality and nutrient provision for bees and protect the ecosystem service of pollination (Dicks et al., 2016). In Europe, land managers are encouraged to develop such environmentally friendly practices for wildlife conservation (Batáry et al., 2015) through financially incentivized Agri‐Environment Schemes (AES). A cornerstone of pollinator‐facing AES is the provision of suitable foraging resources on arable farmland by sowing seed mixtures at the margins of crops in so‐called wildflower strips (WFS). These usually include annual and biennial, or perennial, flowering species that offer pollen and nectar rewards (e.g., https://www.gov.uk/countryside‐stewardship‐grants), with the aim of attracting flower‐visiting insects and subsequently promoting pollination services or biological pest control (Haaland et al., 2011).

A number of studies have shown increased abundance of insect pollinators in response to locally increasing flower provision with WFS (e.g., Carvell et al., 2007, 2011; Lowe et al., 2021), as well as long‐lasting positive effects by enhancing the establishment and persistence of wild bee nests (Carvell et al., 2017; Klatt et al., 2020; Wood, Holland, Hughes, et al., 2015). Though WFS are generally shown to increase pollinator diversity when compared with non‐restored areas (Carvalheiro et al., 2011; Lowe et al., 2021), seed mixes used for WFS often lack flowers suitable for specialist oligolectic species (Wood, Holland, & Goulson, 2015). In the UK for example, WFS were initially designed to meet the foraging requirements of declining bumblebees by including a high proportion of Fabaceae and showed positive effects on both common and threatened bumblebee species (Carvell et al., 2007), but offered limited foraging opportunities for the wider pollinator community (Gresty et al., 2018; Scheper et al., 2013; Wood et al., 2017).

Beyond the increase in bee abundance and diversity, there is only a limited understanding of the effects of changes in flower provision on the structure of plant‐pollinator networks. Kaiser‐Bunbury et al. (2017) showed a diversification of interactions in restored tropical plant‐pollinator networks, as higher plant species richness increased pollinator diet breadth (i.e., the number of visited flower species). Gao et al. (2021) showed moreover that restored networks with more diverse interactions are more stable and robust to perturbations and species loss, as measured by network connectance. In contrast, heathland restoration in England led to a reduction in insect pollinator network connectance, independent of plant species richness (Forup et al., 2007). Overall, it remains unclear how enhanced flower provision modifies pollinator foraging niche partitioning and competition for resources. In agricultural areas, seed mixes for WFS are designed to meet the dietary needs of mostly generalist bees, with high flower density and enhanced flower species richness. One can hypothesize that higher flower species richness will expand the foraging spectrum of pollinators, as observed in Kaiser‐Bunbury et al. (2017) and Gao et al. (2021), and in consequence may increase competition between pollinators when foraging niches overlap. Increasing the number of insect species visiting the same plants by promoting niche overlap and connectance can be beneficial for network robustness (the resilience of networks following the loss of species), and hence is often a goal in pollinator restoration (e.g., Cusser & Goodell, 2013). However, higher niche overlap may lead to direct competition if resources (i.e., nectar and pollen) are limited in space and time (Goulson & Sparrow, 2009; Wignall et al., 2020) and to indirect competition by providing opportunities for the transmission of pathogens (Proesmans et al., 2021).

In this study, we recorded bee‐plant interactions on UK farms implementing different levels of pollinator restoration measures and investigated the effect of increased flower species richness and density on pollinator networks, with a focus on resource exploitation and overlap in bees. We thus measured resource exploitation by bees as the generality index, calculated as the mean number of visited flower species per bee species. We also calculated two network metrics: connectance, a measure of interaction diversity within networks and a relevant index to predict disease transmission between bees (Figueroa et al., 2020), and bees' niche overlap, a measure of resource sharing and a proxy for competition between taxa (Taggar et al., 2021). We focused our study on honeybees and bumblebees, two common and important crop pollinator taxa with a strong potential for competitive interactions (Goulson & Sparrow, 2009; Wignall et al., 2020) and disease transmission (Fürst et al., 2014; Manley et al., 2019; Piot et al., 2022).

We hypothesize that higher flower species richness may increase the number of visited flower species by bees (i.e., the generality index) and in consequence increase both the number of interactions within plant‐pollinator networks (i.e., increased connectance) and the level of shared resources by bees (i.e., increased niche overlap). We discuss the importance of considering these indices when restoring pollinator networks and their implications for pollinator health and competition.

## 2 MATERIALS AND METHODS

### 2.1 Site selection and data collection

**FIGURE 1:**
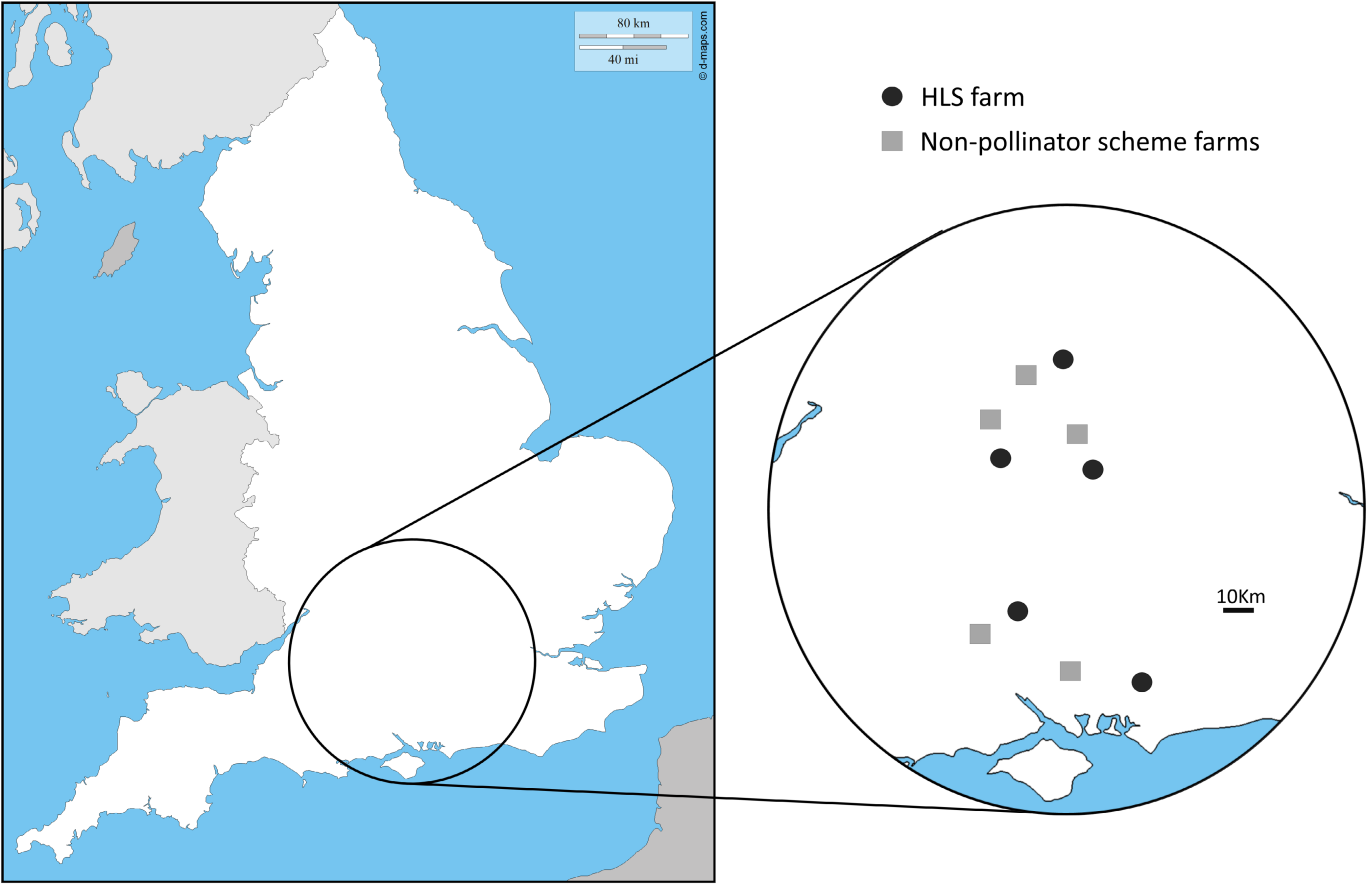
Map of England showing the location of study sites. Black circles show HLS farms; gray squares show farms not participating in pollinator schemes. All farms are at least 10 km apart. Map modified from d‐maps.com.

We performed this study across 10 farms in Southern England, with five farms participating in the Higher Level Stewardship (HLS) AES for pollinators (Natural England, 2013) including WFS across the study area, two farms not participating in pollinator schemes but providing other WFS such as flowering cover crops, game cover, or recreational flower margins (Appendix 1), and three farms with no additional wild‐flowers for pollinators. To ensure independence of data collection, farms were at least 10 km apart (Figure 1), covering the maximum foraging distance of honeybee workers (Steffan‐Dewenter & Kuhn, 2003). We also ensured that farms not applying AES for pollinators were not directly adjacent to other HLS farms using maps from the MAGIC (Multi‐Agency Geographic Information for the Countryside) geoportal (Askew et al., 2005).

We visited each farm at three time points: in early (18th–30th June) and late (30th July–10th August) summer 2016 when WFS were in bloom and during the following spring before the onset of WFS flowering (from 25th March to 9th April 2017). We recorded flower density and richness on two to three transects per farm and time point, depending on the flower provision at the time we performed the survey. Transects of 100 m length and 2 m width were selected for high abundance and richness of flowers and insect visitors within the farm. In farms not involved in pollinator scheme, or when no WFS were flowering on HLS farms, transects were performed on other non‐cropped field margins such as hedgerows. Thus, transect locations were not always the same across seasons but were adjusted as necessary to capture the highest density and richness of flower species. In total, 55 summer transects were performed, including 28 on WFS, and 27 on other field margins with no sown flowers. In spring 2017, we performed transects either on hedgerows including perennial trees and shrubs, on field margins with perennial flowering plants such as *Lamium album*, or on flowering oilseed rape crop fields when no flower resources were available on field margins. The spring floral data do not include flower density as it cannot be reliably estimated for large flowering trees such as *Prunus* sp. and *Salix* sp., which represented important pollinator resources.

For each summer transect, we recorded the number of flower units per species in a 0.25 m^2^ quadrat randomly thrown every 10 m. Flowering units were defined as in Carvell et al. (2007); one flower “unit” was counted as a single flower or, in the case of multiflowered stems, as an umbel, head, spike, or capitulum. Flowering plants were identified to species in most cases, otherwise to the family or genus. Flower species richness and density (mean number of flowering units per quadrat) were calculated for each transect. Plant‐insect interaction sampling consisted of walking 15 min along the entire transect and recording all observed insect visitors that contacted flowers within 1 m on either side of the transect line (O'Connor et al., 2019). Honeybees and bumblebees were identified as species, with the exceptions of the species complexes *Bombus terrestris/lucorum/cryptarum/magnus* and *Bombus hortorum/ruderatus*, neither of which have workers that are readily identifiable on the wing. Before each transect observation, we recorded ambient temperature in the shade, the percentage of cloud cover in the sky, and estimated wind speed following the Beaufort scale. Insect observations were performed only in favorable conditions, including wind speed at a maximum of 5 on the Beaufort scale, and a minimum ambient shade temperature of 15°C in summer and 9°C in spring.

### 2.2 Data analysis

Network metrics were calculated from plant‐insect interactions involving exclusively the honeybee *Apis mellifera* and eight bumblebee taxa: *Bombus hortorum/ruderatus*, *B. hypnorum*, *B. lapidarius*, *B. pascuorum*, *B. pratorum*, *B. rupestris*, *B. terrestris/lucorum/cryptarum/magnus*, and *B. vestalis*. To test the impact of additional flower provision on bees' resource exploitation and overlap, we calculated three network indices using the R package *bipartite* (Dormann et al., 2009). For each transect we computed: (i) bee species' generality index (average number of plant species visited by each bee species), as a measure of foraging choice; (ii) weighted connectance (the realized proportion of possible bee‐plant interactions in the network weighted by the number of observations for each interaction); and (iii) bee species' niche overlap (weighted mean similarity in interaction patterns with flower species among all bee species of a network) calculated as Horn‐Morisita similarity, as a measure of competition between bees. Because generality and niche overlap are sensitive to the number of observed interactions (Nielsen & Bascompte, 2007; Vanbergen et al., 2017), we standardized these two network parameters using *z*‐scores against 5000 random networks following the null model (*vaznull* function) implemented in the *bipartite* package (Vázquez et al., 2007). This function generates binary matrices with randomized interaction probabilities proportional to each species' relative abundance, constrained by the connectance of the original network. Because *z*‐scores cannot be generated on small networks, spring transects, which all included too few flower and bee species, and three summer transects with only two or fewer plant species involved were discarded for the analyses of network metrics. The resulting *z*‐scores were used in the statistical models below. As null models were constrained by connectance, statistical tests were performed directly on the weighted connectance, rather than calculating *z*‐scores for this measure.

We compared flower species richness, flower density, and abundance of honeybees plus bumblebees between WFS and other field margins using *t*‐tests. Flower species richness was measured as flowering plant species visited at least once by any insect pollinator as recorded in our transects (i.e., visits included, but were not limited to, honeybees and bumblebees). After verifying that species richness and flower density were normally distributed and after natural log transforming bee abundance data to meet normality, we performed Student's *t*‐tests when variance was equal between the two tested categories and Welch's *t*‐tests when variance was unequal. To test the effect of flower density and flower species richness on bee abundance, we applied a generalized linear mixed model with Poisson distribution where environmental data (temperature, wind scale, and cloud cover) and sampling period were used as fixed variables and farms as a random variable, plus an observation‐level random effect to account for overdispersion (Harrison, 2014). We started with the most complex model including a three‐way interaction between flower density, flower species richness, and sampling period, as flower density and richness vary across time and may influence bee abundance. We then simplified models for a better fit by model selection using *F*‐tests and Akaike information criterion (AIC). To test the effect of flower density and flower species richness on network metrics, we performed linear mixed models where environmental data and sampling period were used as fixed variables and farms as a random variable. To disentangle the interaction effects of the fixed variables, flower density, flower species richness, and sampling periods, similar models were applied for each sampling period. We checked overdispersion in regression analysis using the function overdisp_fun and multicollinearity using the function vif from the *car* package (Fox & Weisberg, 2019). GLMMs were run using the R package *lme4* (Bates et al., 2014) while LMMs were run with *blme* (Chung et al., 2013). Continuous variables, namely flower density, flower species richness, and environmental variables were centered for use in mixed models. We considered .05 as a significant threshold for *p* values, while *p* values between .06 and the threshold were considered marginally significant.

## 3 RESULTS

### 3.1 Flower richness and density

**FIGURE 2:**
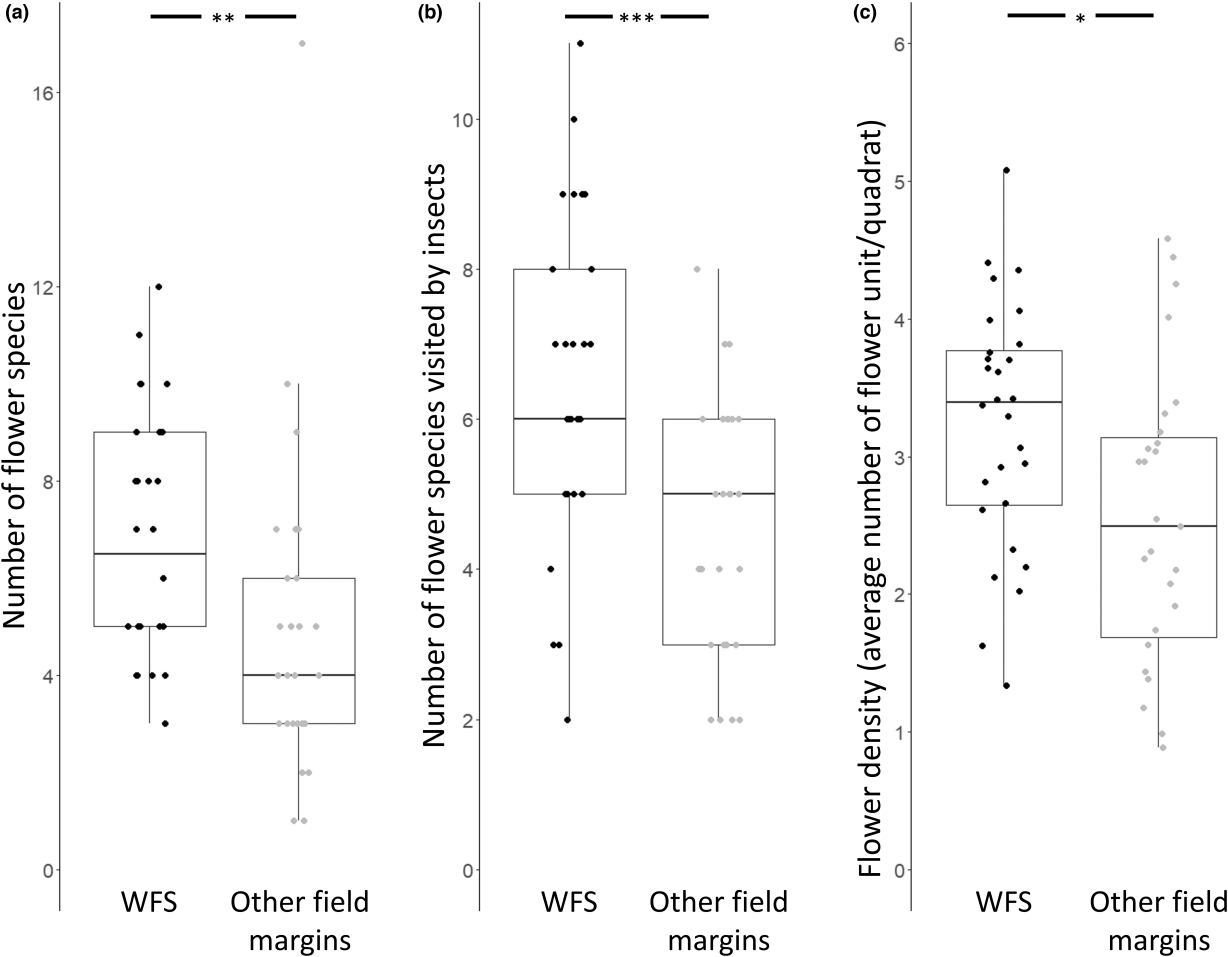
Box plots showing (a) higher flower species richness, (b) higher number of insect‐visited flower species, and (c) higher flower density (ln‐transformed values) in wildflower strips (WFS, black) in comparison to other field margins (gray). Data collection from two time points in summer 2016. *p < .05, **p < .01, ***p < .001.

**FIGURE 3:**
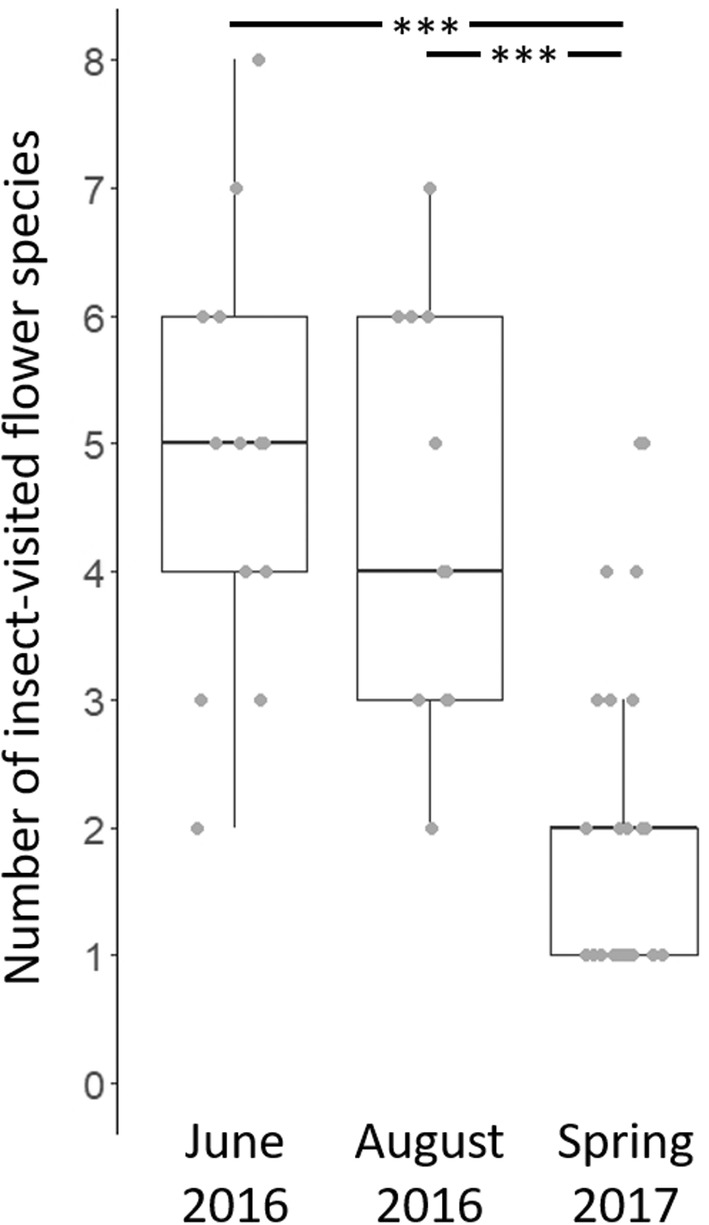
Box plots illustrating the higher number of insect‐visited flower species observed in summer (June and August 2016) compared to the following spring (March–April 2017) in the same farms (on non‐WFS habitats), with median and 95% confidence intervals. ***p < .001.

Total flower species richness was higher on WFS in comparison to other field margins (average richness (±SEM) for WFS = 6.8 (±0.5); others = 4.9 (±0.6); *t* = −3.14, *p* = .003; Figure 2a), as was the number of insect‐visited flower species (WFS = 6.4 (±0.4); others = 4.5 (±0.3); *t* = −3.578, *p* < .001; Figure 2b). WFS also supported a higher flower density (WFS = 36.3 (±6.2) flower units per quadrat; others = 21.8 (±5); *t* = −2.672, *p* = .01; Figure 2c). Seed mixes for WFS changed the overall floral composition of agricultural landscapes, as 33% and 66% of total flower species were uniquely recorded on WFS in June and August 2016, respectively (Appendix 2). When compared to spring, the total number of insect‐visited flower species in non‐WFS transects was much higher during summer (June 2016 vs. Spring 2017 *t*(51) = −6.357, *p* < .001; August 2016 vs. Spring 2017 *t*(51) = −4.906, *p* < .001; Figure 3).

### 3.2 Insect visits to flowers

**FIGURE 4:**
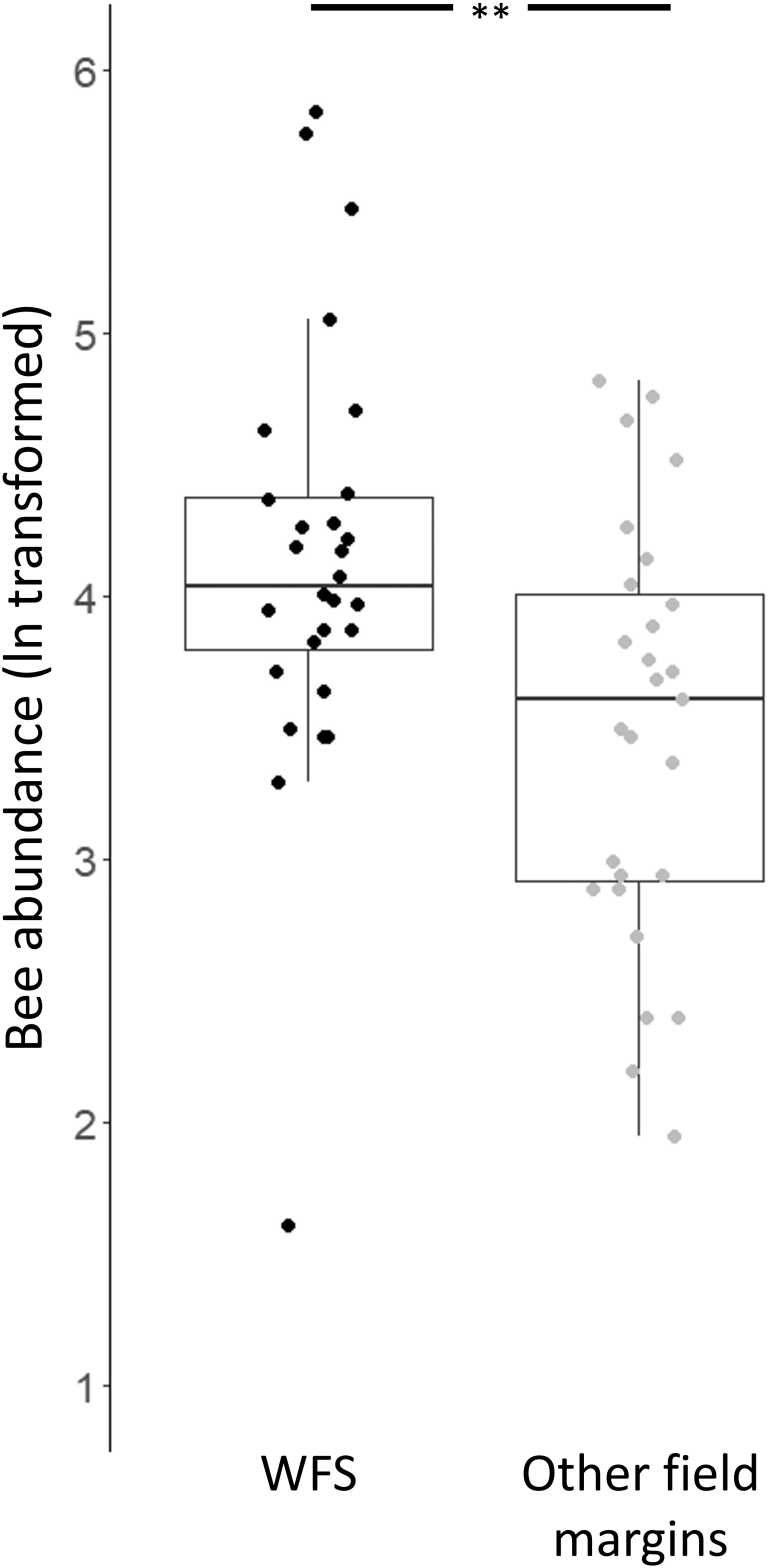
Box plots showing ln‐transformed total numbers of honeybees and bumblebees recorded on transects, with median and 95% confidence intervals. Black dots and box represent WFS, gray dots and box represent other field margins. Data collection from two time points in summer 2016. **p < .01.

In total, we recorded 5865 interactions during 825 min of summer transect observations. Of these, 3673 were between bees and flowers, including 1248 honeybees (34.0% of all bees) and 2330 bumblebees (63.4%) representing eight *Bombus* taxa. We observed more bees on WFS than on other field margins (average per transect: WFS = 85.6 (±15.6); others = 43.7 (±6.4); *t*(53) = −2.916, *p* = .005; Figure 4), this effect being mainly driven by bumblebees (*t*(53) = −3.8619, *p* < .001) rather than honeybees (*t*(53) = −0.636, *p* = .528; Appendix 3). Analysis across transects showed that time points, cloud cover, and the interaction between time point and flower density were significant factors influencing bee abundance (Appendix 4). To disentangle the interaction effect, we ran separate models for June and August 2016. In June, flower density had no significant effect on bee abundance, but higher flower species richness significantly increased bee abundance (Table 1A). In August, higher flower density significantly increased bee abundance (Table 1B). Models showed neither overdispersion nor multicollinearity among variables (Table 1 and Appendix 4).

**TABLE 1:** Estimated regression parameters, standard errors, *Z*‐values, and *p*‐values for the Poisson GLMM performed for bee (honeybee and bumblebee) abundance, from observation of June 2016 (A) and August 2016 (B) on WFS and other field margins.

	Estimate	Std. error	*Z*‐value	*p*‐Value	VIFs
*(A) GLMM for bee abundance in June 2016*					
Intercept	3.459	0.201	17.244	**<.001**	–
Flower density	0.000	0.005	−0.093	.926	1.247
Flower species richness	0.116	0.057	2.052	**.040**	1.266
Temperature	0.015	0.081	0.185	.854	1.228
Wind speed	0.130	0.114	1.142	.254	1.175
Cloud cover	0.002	0.005	0.323	.747	1.163
*(B) GLMM for bee abundance in August 2016*					
Intercept	4.299	0.122	35.342	**<.001**	–
Flower density	0.014	0.002	7.086	**<.001**	1.061
Flower species richness	0.007	0.036	0.184	.854	1.133
Temperature	0.014	0.036	0.381	.703	1.036
Wind speed	−0.046	0.096	−0.478	.632	1.105
Cloud cover	−0.006	0.003	−1.922	.055	1.116

*Note*: Significant *p*‐values are shown in bold characters. Fraction of the variance explained: R2m = 0.145 and R2c = 0.949 for (A) and R2m = 0.598 and R2c = 0.973 for (B). Overdispersion tests *χ* ^2^ = 2.968, *p* = 1 for (A) and *χ* ^2^ = 4.590, *p* = 1 for (B). Variance Inflation Factors (VIFs) show no correlation between variables (i.e., no VIF > 5).

### 3.3 Generality, weighted connectance, and niche overlap

**FIGURE 5:**
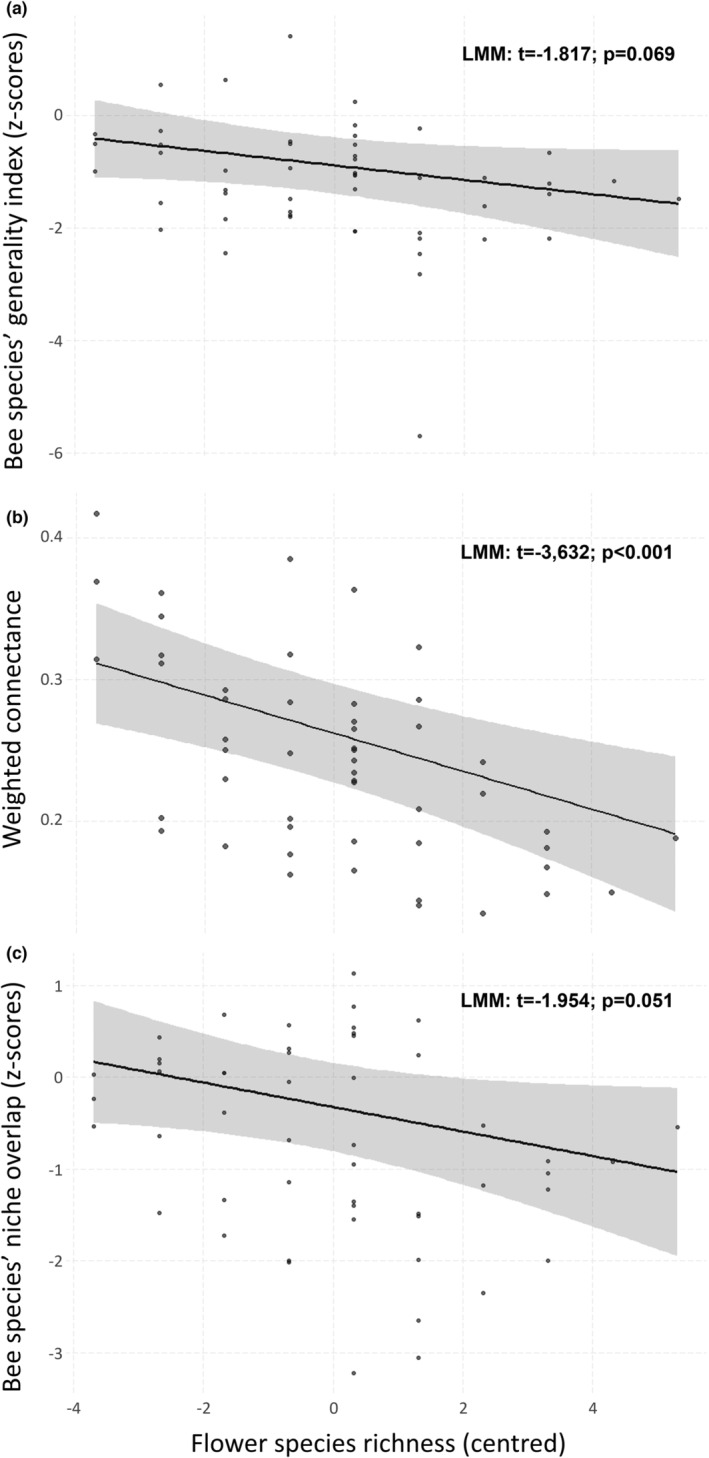
Scatter plots showing the relationship between flower species richness and network metrics: (a) bee species' generality indices, (b) weighted network connectance, and (c) niche overlap across honeybees and eight *Bombus* species. Plotted lines show the estimated effects, and shaded areas indicate the 95% confidence intervals as predicted by LMMs. Dots represent transects' network indices from our two data collection time points of summer 2016.

Flower species richness had no significant effect on bee species' generality indices (*t* = −1.816; *p* = .069; Figure 5a), but had a significant negative effect on network connectance (*t* = −3.615; *p* < .001; Figure 5b), and a negative but non‐significant effect on bee species' niche overlap (*t* = −1.952; *p* = .051; Figure 5c). Flower density had no significant effect on the three‐network metrics (Appendix 4): bee species’ generality indices (*t* = 0.103; *p* = .918), weighted connectance (*t* = −0.349; *p* = .727), and bee species’ niche overlap (*t* = −0.772; *p* = .440). We found a significant effect of collection time on bee species’ niche overlap (*t* = −2.010; *p* = .044) and weighted connectance (*t* = −2.016; *p* = .044), with a lower niche overlap and weighted connectance in August networks compared to June, and a significant positive effect of wind speed on weighted connectance (*t* = 2.083; *p* = .037). Models showed neither overdispersion nor multicollinearity among variables (Appendix 4).

## 4 DISCUSSION

Manipulation of flower provision has been widely used for insect pollinator conservation in agricultural landscapes and to restore the ecosystem service of pollination, including via the establishment of sown wildflower strips (WFS) (Haaland et al., 2011; Scheper et al., 2013). Despite their implementation for almost three decades, the impact of increased flower density and richness on the structure of plant‐pollinator networks has been largely ignored. In contrast to our original hypothesis, our study demonstrates that increasing flower species richness does not significantly change bees' diet breadth (as measured by generality), but does reduce network connectance (the proportion of realized interactions within networks), and marginally reduces bees' niche overlap, a measure of shared resources and a proxy for competition. Our results suggest that increasing flower species richness in agricultural areas may provide unexpected benefits for the bee community by partitioning bee species’ foraging niche and potentially reducing competition for resources.

We found that bumblebee abundance was significantly increased in WFS in comparison to other non‐cropped field margins. Our results confirm a general trend from several studies across a range of agricultural landscapes and countries where WFS elevated insect pollinator observations (e.g., Carvell et al., 2007, 2011; Lowe et al., 2021). Honeybees are domesticated insects and their abundance is largely influenced by beekeeping activities (Valido et al., 2019). Accordingly, we showed that higher bee abundance on WFS was mainly driven by a significant increase in bumblebee abundance. Higher abundance of bees along field margins with WFS has been associated with increased pollination service in surrounding crops (e.g., Blaauw & Isaacs, 2014; Carvalheiro et al., 2012; Ganser et al., 2018; Pywell et al., 2015), but not always (e.g., Albrecht et al., 2020; Delphia et al., 2022). Importantly, we identified flower density as a major variable contributing to the higher abundance of bees in August, in accordance with Carvell et al. (2007). Optimal foraging theory predicts higher rates of pollinator visits on denser patches of flowers, maximizing the net rate of energy intake per foraging trip (Pyke et al., 1977). Our data support the principle that higher flower density is necessary to support bee foraging, and that WFS must provide continuous dietary resources each year across the pollinator season (Schellhorn et al., 2015). Higher flower species richness also increased bee abundance in June. The higher abundance of honeybees and bumblebees on transects with high flower species richness may have been caused by colony numbers increasing towards mid‐summer, and therefore more bees being on the wing foraging at this time. High flower diversity is generally linked to higher insect diversity (Ebeling et al., 2008; Hudewenz et al., 2012; Lane et al., 2020; Potts et al., 2003). However, as our analysis was restricted to honeybees and bumblebees, with a maximum of seven species observed over a single transect, we were unable to investigate the effect of higher flower species richness on bee species richness.

Currently, many WFS in England are not designed to provide nectar and pollen resources early in the season (Wood et al., 2017), although spring is the critical period for bumblebee nest establishment. Flower supply in early spring is an important factor in securing the establishment of colonies and increasing the reproductive success of wild bumblebee populations (Carvell et al., 2017; Holzschuh et al., 2016). With our survey, we showed that flower richness in spring is dramatically reduced in comparison to summer, being largely limited to mass‐flowering crops, spring‐flowering trees, and hedgerow species (e.g., *Prunus* sp., *Salix* sp., and *Lamium* sp.). This reduction in flower species richness likely results in the concentration of all bee species on a few resources, increasing niche overlap and potentially increasing competition, as observed in summer (see below). There are potential measures that may provide alternatives or additions to traditional WFS to promote the early establishment of bee populations. Annual WFS seed mix options, such as spring‐flowering WFS or the autumn‐sown “bumblebird” mixture whose primary function is to provide winter food for seed‐eating farmland birds, can support the provision of uninterrupted food resources for pollinators across the season (Carvell et al., 2006; Natural England, 2017). One of our sampling sites, with a game cover crop seed mixture, dominated by *Brassica oleracea*, was highly attractive to honeybees and queen bumblebees in spring. In late summer, cover crop strips (i.e., flowering species planted mainly for the purpose of protecting or improving the soil, and enhancing biodiversity, with no intention of harvesting) dominated by unsown wildflowers such as *Sonchus arvensis*, *Cirsium vulgare*, or *Epilobium hirsutum* also provided excellent sources of forage for honeybees and bumblebees. Encouraging the broader adoption of such alternatives to traditional WFS may be instrumental in providing forage resources across the pollinator season.

Changes in flower provision also altered the structure of plant‐pollinator networks. For instance, we found that higher flower species richness marginally reduced foraging niche overlap across honeybee and bumblebee species. This suggests that bees partitioned their niche by visiting different plant species. This result is particularly relevant for conservation perspectives as niche overlap can be used as a proxy for resource competition (Taggar et al., 2021). Exploitative competition for floral resources occurs when the consumption of limiting floral resources overlaps between species, leading to a shift in floral species use (Magrach et al., 2017), or resulting in a potential reduction of insect population size, fecundity, or survival, for at least one of the interacting species (Thomson, 2004).

Interestingly, providing more flower‐rich patches did not significantly increase diet breadth, measured here as generality among bee species. Many studies showed that increasing flower species richness promotes a diverse diet for bees (Baldock et al., 2015; Gao et al., 2021; Kaiser‐Bunbury et al., 2017). However, the design of seed mixes for WFS in England has been typically tailored for bumblebees (Carvell et al., 2007) and this may have resulted in no increase in diet breadth, as it is not surprising to observe bees foraging on their preferred flowers once they are provided with a choice. It is important to note that the combined records for the species complexes *Bombus terrestris/lucorum/cryptarum/magnus* and *Bombus hortorum/ruderatus* may lead to slight overestimates for our indices, as species within these complexes may have different foraging preferences.

The negative effect of flower species richness on niche overlap could be driven by the diverse dietary requirements among bees (Kriesell et al., 2017; Vaudo et al., 2016) and their preference for different flower morphologies (Inouye, 1980). Alternatively, bees may have modified their foraging spectrum and behaviors in response to apparent competition (Stephens & Krebs, 1986). For instance, pollinators tend to distribute their foraging effort towards less‐connected flower species in flower‐rich habitats, an adaptive foraging behavior that leads to niche partitioning (Valdovinos et al., 2016). The apparent reduction of pollinators' niche overlap with increasing flower abundance observed by Tommasi et al. (2021) in sub‐Saharan farms also supports this hypothesis.

Niche partitioning could also have been a consequence of a reduction in network connectance in our study, measured as the proportion of realized interactions between flower and bee species. Although we expected the addition of flower species tailored for bumblebee dietary requirements to increase network connectance, it is well documented that network connectance decreases when the number of nodes increases (Dormann et al., 2009), including in plant‐pollinator networks (Basilio et al., 2006; Olesen & Jordano, 2002). Accordingly, we found significantly decreased network connectance with increased flower species richness. Keeping high network connectance often remains a desired outcome for pollinator restoration (e.g., Cusser & Goodell, 2013), as high connectance (and hence a high degree of generalism and high niche overlap) is believed to confer greater resilience to species loss (Dunne et al., 2002; Thébault & Fontaine, 2010).

Variation in connectance, and other network parameters such as nestedness and modularity, is also known to influence the transmission dynamics of pathogens and parasites within host networks (Proesmans et al., 2021; Shirley & Rushton, 2005). For instance, high connectance of pollinator networks has recently been associated with a reduction of pathogen prevalence in bees (Figueroa et al., 2020), potentially due to the so‐called dilution effect, that is, a reduction in successful transmission to susceptible hosts over a diversity of plant‐pollinator interactions. Honeybees and bumblebees notoriously share many pathogens such as viruses, fungi, and eukaryotes (Manley et al., 2015). Accumulating evidence from phylogenetic studies shows that pathogen genotypes are shared between species within a population, suggesting that interspecific transmission may be a common mechanism in bees (Fürst et al., 2014; Manley et al., 2019, 2020), which is believed to take place via shared floral use (Adler et al., 2020; Durrer & Schmid‐Hempel, 1994). Thus, parameters such as connectance and niche overlap inform us about the host “contact network” defined by the level of shared resources by pollinators (Wilfert et al., 2020). Hence, diversifying flower traits and increasing flower abundance can reduce interspecific disease transmission in bees, by promoting niche partitioning (Adler et al., 2020) or diluting foragers in pollinator networks (Graystock et al., 2020), respectively. Here, we showed that increasing flower species richness in farmland habitats can reduce pollinator network connectance and may also reduce niche overlap across honeybee and bumblebee species. By increasing floral diversity and incorporating plant characteristics in the design of WFS seed mixes, the potential of these conservation measures to impact disease spread and safeguard bee health could be maximized. However, whether this leads to a reduction in pathogen transmission via flowers will need to be verified by empirical studies in the field.

## 5 CONCLUSION

In this study, we showed that sown wildflower strips (WFS) can provide multiple benefits for bees. We demonstrated that higher flower density in WFS is a key factor in attracting honeybees and bumblebees, while higher flower species richness provides a diversified diet in comparison to otherwise florally deprived agricultural landscapes. More importantly, we identified a potential unexpected beneficial effect of increased flower diversity on the pollinator community. We showed that increasing flower species richness reduces connectance and marginally reduces niche overlap and, as a result, has the potential to reduce competition for resources and alter disease transmission between managed and wild bee species. While current practices for pollinator conservation promote network connectance and niche overlap to improve network robustness (Cusser & Goodell, 2013; Devoto et al., 2012; Menz et al., 2011), our study demonstrates that careful design of WFS seed mixes may provide good forage to bees while promoting moderate niche overlap and prevent direct and indirect competition between insect pollinators.

Finally, these results argue for an extension of measures to provide diverse foraging resources into the crucial spring period. An increase of flower density and diversity in spring should provide similar benefits to those observed in summer, that is, the provision of a diverse and abundant diet, as well as a potential reduction in competition and a drop in inter‐species disease transmission. Such measures promise to improve the abundance, diversity, and health of insect pollinators, with the wider benefit of restoring farmland biodiversity and ecosystem services, but highlight the urgent need for research testing these effects in realistic field scenarios.

## AUTHOR CONTRIBUTIONS

**Vincent Doublet:** Conceptualization (equal); data curation (equal); formal analysis (lead); investigation (equal); methodology (equal); visualization (lead); writing – original draft (lead); writing – review and editing (equal). **Toby Doyle:** Conceptualization (equal); data curation (equal); investigation (equal); writing – review and editing (equal). **Isobel Refoy:** Investigation (supporting); writing – review and editing (equal). **Sophie Hedges:** Investigation (supporting); writing – review and editing (equal). **Claire Carvell:** Conceptualization (equal); investigation (equal); methodology (equal); resources (equal); writing – review and editing (equal). **Mark J. F. Brown:** Conceptualization (lead); funding acquisition (equal); methodology (equal); project administration (equal); resources (equal); writing – review and editing (equal). **Lena Wilfert:** Conceptualization (lead); funding acquisition (equal); methodology (equal); project administration (equal); resources (equal); supervision (lead); writing – original draft (equal); writing – review and editing (equal).

## FUNDING INFORMATION

This work was supported by the Biotechnology and Biological Sciences Research Council (BBSRC) under grants BB/N000668/1 and BB/N000625/1 to MJFB and LW, respectively. The contribution of CC was supported by the Natural Environment Research Council (NERC) under research program NE/N018125/1 ASSIST – Achieving Sustainable Agricultural Systems www.assist.ceh.ac.uk. ASSIST is an initiative jointly supported by NERC and BBSRC. Open Access funding enabled and organized by Projekt DEAL.

## CONFLICT OF INTEREST

None declared.

## Data Availability

Raw data are available on FigShare (https://doi.org/10.6084/m9.figshare.c.5852994.v1).

## APPENDIX 1

Pictures showing a sown wildflower strip (WFS) from a High‐Level Stewardship (HLS) farm (top) and a cover crop habitat (also considered as WFS) from a farm not involved in a pollinator conservation scheme (bottom). Both pictures were taken in Hampshire, UK, in August 2016.

## APPENDIX 2

Venn diagrams showing overlaps in flowering plant species observed between habitats (WFS vs. other field margins) in summer 2016.

## APPENDIX 3

Box plots showing ln‐transformed total numbers of honeybees and bumblebees separately recorded on transects, with median and 95% confidence intervals. Black dots and box represent WFS, gray dots and box represent other field margins. Data collection from two time points in summer 2016. “ns” for non‐significant and ****p* < .01.

## APPENDIX 4

Estimated regression parameters, standard errors, *Z*‐values, and *p*‐values for the Poisson GLMM performed for bee abundance, from all summer observations on WFS and other field margins. See also Table 1A and B, where this analysis is decomposed into two separate models for June 2016 and August 2016. Significant *p*‐values are shown in bold characters. Fraction of the variance explained: R2m = 0.598 and R2c = 0.973. Overdispersion tests *χ* ^2^ = 6.633, *p* = 1. Variance Inflation Factors (VIFs) show no correlation between variables (i.e., no VIF > 5).

**Table:**

	Estimate	Std. error	*Z*‐value	*p*‐Value	VIFs
Intercept	3.466	0.374	25.23	**<.001**	–
Flower density	0.002	0.005	0.441	.659	3.314
Flower species richness	0.039	0.038	1.041	.298	1.185
Time points	0.779	0.175	4.461	**<.001**	1.572
Temperature	0.037	0.041	0.897	.370	1.240
Wind speed	0.000	0.074	0.001	.999	1.362
Cloud cover	−0.006	0.003	−2.269	**.023**	1.131
Flower density × time points	0.015	0.005	2.707	**.007**	3.238

Estimated regression parameters, standard errors, *t*‐values, and *p*‐values for the LMM performed for bees' generality index, from all summer observations on WFS and other field margins. Fraction of the variance explained: R2m = 0.183 and R2c = 0.215. Overdispersion tests *χ* ^2^ = 43.452, *p* = .409. Variance Inflation Factors (VIFs) show no correlation between variables (i.e., no VIF > 5).

**Table:**

	Estimate	Std error	*t*‐Value	*p*‐Value	VIFs
(Intercept)	−0.887	0.248	−3.581	.000	–
Flower species richness	−0.128	0.071	−1.817	.069	1.063
Flower density	0.001	0.006	0.108	.914	1.138
Time point	−0.599	0.366	−1.635	.102	1.692
Cloud cover	−0.002	0.005	−0.359	.719	1.134
Temperature	−0.109	0.079	−1.387	.166	1.203
Wind speed	0.089	0.128	0.695	.487	1.330

Estimated regression parameters, standard errors, *t*‐values, and *p*‐values for the LMM performed for weighted connectance, from all summer observations on WFS and other field margins. Low *p*‐values are shown in bold characters. Fraction of the variance explained: R2m = 0.309 and R2c = 0.614. Overdispersion tests *χ* ^2^ = 0.079, *p* = 1. Variance Inflation Factors (VIFs) show no correlation between variables (i.e., no VIF > 5).

**Table:**

	Estimate	Std error	*t*‐Value	*p*‐Value	VIFs
(Intercept)	0.262	0.017	15.192	<.001	‐
Flower species richness	−0.013	0.004	−3.632	**<.001**	1.079
Flower density	0.000	0.000	−0.283	.775	1.157
Time point	−0.036	0.018	−1.998	**.046**	1.870
Cloud cover	0.000	0.000	0.794	.427	1.148
Temperature	−0.004	0.004	−1.055	.291	1.261
Wind speed	0.018	0.009	2.070	**.038**	1.543

Estimated regression parameters, standard errors, *t*‐values, and *p*‐values for the LMM performed for bees' niche overlap, from all summer observations on WFS and other field margins. Significant *p*‐values are shown in bold characters, and marginally significant *p*‐value is shown in italic. Fraction of the variance explained: R2m = 0.215 and R2c = 0.246. Overdispersion tests *χ* ^2^ = 40.546, *p* = .535. Variance Inflation Factors (VIFs) show no correlation between variables (i.e., no VIF > 5).

**Table:**

	Estimate	Std error	*t*‐Value	*p*‐Value	VIFs
(Intercept)	−0.322	0.239	−1.349	.177	–
Flower species richness	−0.133	0.068	−1.954	*.051*	1.063
Flower density	−0.005	0.006	−0.767	.443	1.138
Time point	−0.711	0.354	−2.011	**.044**	1.692
Cloud cover	−0.001	0.005	−0.288	.773	1.134
Temperature	−0.093	0.076	−1.228	.220	1.203
Wind speed	0.123	0.123	0.995	.320	1.330
